# Comparing the effectiveness of an e-learning module at different levels of magnification for detecting occlusal caries in permanent teeth, utilizing the international caries detection and assessment system (ICDAS): an ex vivo study

**DOI:** 10.1038/s41405-025-00323-x

**Published:** 2025-04-28

**Authors:** Mary Byju, Kundabala Mala, Srikant Natarajan, Manuel S. Thomas, Abhishek Parolia

**Affiliations:** 1 Private practitioner, Root canal home, Family Dental Clinic, Vandanam, Alappuzha, Kerala 688005 India; 2 Department of Conservative Dentistry and Endodontics, Manipal College of Dental Sciences Mangalore, Affiliated to Manipal Academy of Higher Education, Manipal, Karnataka 576104 India; 3 Department of Oral Pathology, Manipal College of Dental Sciences Mangalore, Affiliated to Manipal Academy of Higher Education, Manipal, Karnataka 576104 India; 4 Department of Endodontics, University of Iowa College of Dentistry and Dental Clinics, Iowa City, Iowa 52242 USA

**Keywords:** Caries risk assessment, Diseases

## Abstract

**Objective:**

The purpose of this study was to compare the effectiveness of low and high magnifications in detecting occlusal caries in permanent posterior teeth using the International Caries Detection and Assessment System (ICDAS) II criteria through the ICDAS e-learning program.

**Materials and methods:**

Forty extracted permanent posterior teeth were used. Two examiners received e-learning training on the ICDAS webpage for detecting occlusal caries before visual examinations. Visual inspections were performed under ×2.5 magnification with a magnifying loupe and ×25 magnification with a dental surgical microscope within a week. The ICDAS scores assigned by both examiners were validated by an ICDAS expert. The visual assessment results were compared with the histological analysis results using Downer’s criteria as the gold standard.

**Results:**

Inter-examiner reliability for ICDAS-II scoring at low magnification (×2.5 loupe) and high magnification (×25 operating microscope) was moderate. However, the lenient (binary) criteria showed substantial agreement at both magnifications. Specificity was highest for both the low and high treatment thresholds (88.2% and 81.5%, respectively) at low magnification, whereas sensitivity peaked at high magnification for the high treatment threshold criterion (91.7%). Image-based ICDAS scoring by an expert also demonstrated good diagnostic accuracy (76.9%), though it was not superior to ICDAS scoring performed under ×2.5 magnification (82.1%).

**Conclusion:**

The ICDAS-II score, particularly the lenient criterion, demonstrated a strong correlation with histological depth. The e-learning program effectively equips dentists with diagnostic skills. Extreme magnification resulted in the overestimation of dental caries, whereas low magnification (2.5x) resulted in greater diagnostic accuracy.

## Introduction

Accurate detection and assessment of dental caries is critical in clinical dentistry for early intervention, preservation of tooth structure and development of appropriate treatment plans tailored to individual patient needs. Early detection of dental caries will aid in choosing a more conservative approach for treating the lesion [1]. Detecting carious lesions occurring on the occlusal pits and fissures of permanent posterior teeth can be challenging because there could be several variations in the morphology of occlusal pits and fissures. Moreover, the widespread use of fluoride has increased the number of hidden caries that spread in dentin, leading to the challenge of accurately detecting the extent of the lesion [2].

The primary means to detect caries is a thorough visual examination. However, this is a subjective method that is dependent on the examiner’s skill and experience. Therefore, the reliability and accuracy of basic visual inspection for detecting caries are poor. Hence, to improve the accuracy, reliability, and reproducibility of visual assessment systems, various detailed visual assessment systems have been proposed [3]. One such proven visual caries assessment system that correlates the clinical status of teeth with histopathological status is the International Caries Detection and Assessment System-II (ICDAS-II) [3, 4].

Good vision is a prerequisite for identifying visual changes as well as microcavities on the tooth surface when detecting and categorizing dental carious lesions in the ICDAS [5]. Magnifying aids have been used in dentistry to enhance visual acuity. However, there is no clear consensus on the preferred level of magnification to improve the diagnostic outcome using the ICDAS-II. A magnification of up to ×2.5 was found to be effective at detecting dental caries in a recent study by Blumer et al. [6] Another study by Neuhaus and colleagues [5] showed that larger magnifications (×4.8 and ×10) can be misleading and should not be used in visual caries detection. However, a study by Akarslan et al. [7] showed that the use of a microscope at ×16 magnification did not differ from that of unaided vision in restorative treatment decision-making on occlusal surfaces. In addition to magnification, the experience of the dentist has varied with respect to the accuracy of visual caries diagnosis [8–10]. Various studies have reported varying outcomes in caries detection, emphasizing the critical need for accurate diagnosis to enable clinicians to provide appropriate treatment. An ideal clinical tool for caries detection should be user friendly, time efficient, cost effective, and precise, ensuring optimal care delivery [11]. In light of these requirements, the present study was undertaken to evaluate the efficacy of clinical diagnosis via the International Caries Detection and Assessment System (ICDAS) criteria under different magnification levels, which were validated against the actual underlying disease process through histopathological evaluation. This study aimed to test the following null hypotheses: (i) the e-learning module utilizing the ICDAS facilitates clinical caries diagnosis with accuracy comparable to that of histological diagnosis, and (ii) there is no difference in the accuracy of occlusal caries detection in permanent teeth between magnifying loupes (×2.5) and surgical microscopes (×25).

## Materials and methods

### Study design and ethical clearance

The ex vivo study was conducted on extracted human teeth following approval from the Institutional Ethics Committee (protocol reference number: 15120).

#### Sample size

Based on Table 3 of the article by Neuhaus et al. [5], the 95% confidence intervals (CI) are 0.60–0.79 for loupes and 0.51–0.64 for microscopes. At an alpha error of 5% and a power of 90%, the Z-score for the chosen alpha error (Z(1-α/2)) is 1.96, while the Z-score for the chosen power (Z(1-β)) is 1.28. With an average standard deviation of 0.08, a minimum clinically relevant difference of 0.06 results in a required sample size of 38 per group. To account for potential tooth loss during experimentation, the sample size was rounded up to 40 per group.

### Sample selection

A total of 40 recently extracted permanent posterior teeth were selected from a collection of extracted teeth and stored in 10% neutral buffered formalin. The samples included teeth with non-cavitated lesions characterized by discoloration at the deeper enamel level or extending through the enamel, as well as cavitated lesions involving the dentin, provided that there was no pulp exposure. Teeth with occlusal surfaces containing pit and fissure sealants, restorations, hypoplastic pits, or fractures were excluded from the study.

### Sample preparation

The teeth were thoroughly rinsed in sterile water after being cleaned with pumice and water slurries to remove any debris. Digital images of the occlusal surface of the selected teeth were taken via a digital, single-lens reflex, AF/AE camera with a built-in flash (Canon EOS 1300D DSLR camera).

### Examiner selection and training

Two examiners with no visual acuity concerns and over three years of clinical experience using operating microscope, but no prior knowledge of ICDAS scoring, were selected. They underwent *e*-learning training available on the ICDAS webpage for the detection of occlusal caries [12]. The examiners received training in ICDAS scoring through a complimentary ninety-minute e-learning program developed by the ICDAS Foundation. A general overview of the criteria and codes to follow is provided in this course, as well as an explanation of the ICDAS examination protocol and a review of the coding system for examiners. An ICDAS expert with more than 8 years of experience using the ICDAS criteria for caries detection was invited to remotely participate in the validation of the samples via digital images.

### Visual examination protocol

Examinations were conducted via 2 levels of magnification per *e*-learning program via an air/water syringe and a ball-ended probe on the teeth that were presoaked in water. When the examiners coded the teeth, they were advised to use a 3-way air spray syringe to dry them. A Community Periodontal Index of Treatment Needs (CPITN) probe (GDC, India) was provided to each examiner, who was advised not to cause surface damage to the samples while probing. Separate evaluation sheets were provided to each examiner prior to visualization with reference to the International Classification of Disease Scale (ICDAS) scoring criteria (Table 1) [12, 13]. The examinations for the ICDAS criteria were first performed with ×2.5 magnification dental loupes with a working distance of 300 mm and a field of view of 67 mm under LED illumination (Carl Zeiss EyeMag Smart 2.5x loupe, Germany). The teeth were kept hydrated between the examinations. Further examination was carried out one week later using a dental operating microscope (Zeiss OPMI Pico, Germany) at its highest magnification of 25X. The examiners’ scores were verified by an ICDAS expert for authenticity. The ICDAS score was further subcategorized into a binary score as teeth requiring “no restoration” and teeth requiring “restoration”. This was considered the lenient scoring criterion. For the low threshold binary score (I1), an ICDAS score above 1 was kept as the cutoff value for the presence of caries needing restorative treatment, and for the high threshold binary score (I2), the cutoff value was 2 (Table 1) [14]. The reproducibility of the intra-examiner and inter-examiner reproducibility and the reliability of the ICDAS were tested via Cohen’s kappa coefficient. The average of the two examiners’ scores was calculated and compared with the histological analysis.

**Table 1:** ICDAS-II scoring criteria for pit and fissure carious lesions and modifications.

Score	Criteria	ICDAS II low treatment threshold (I1)	ICDAS II high treatment threshold (I2)
0	Sound tooth surface	No intervention	No intervention
1	First visual change in enamel		
2	Distinct visual change in enamel	Active intervention	
3	Localized enamel breakdown due to caries with no visible dentin or underlying shadow		Active intervention
4	Underlying dark shadow from dentin with or without enamel breakdown		
5	Distinct cavity with visible dentin		
6	Extensive distinct cavity with visible dentin		

### Histological analysis

**Fig. 1:**
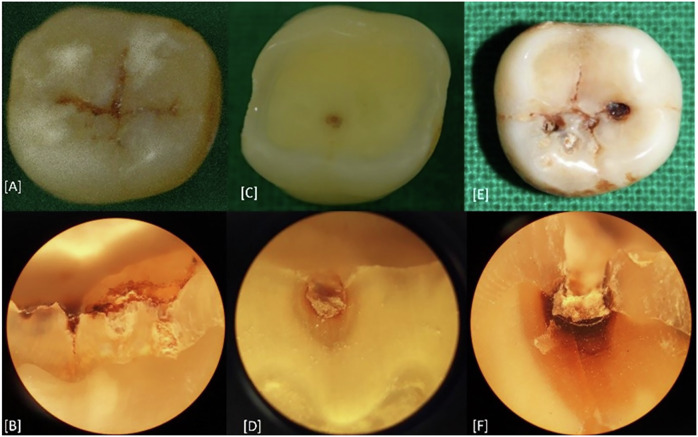
Carious lesions on occlusal surface and their corresponding histological sections. Representative clinical images (**A**, **C**, **E**) and corresponding histological images (**B**, **D**, **F**) of occlusal caries. Histological section (**B**) represents Score 1; **D** represents Score 2 and **F** represents Score 3 based on Downer's histological scoring criteria.

Using 0.15 mm thick diamond abrasive discs (Fein Power Tools India Pvt. Ltd.) mounted on a low-speed motor with water irrigation, the roots of the teeth were resected just apical to the cement‒enamel junction. The tooth samples were mounted in dental plaster. Each tooth was prepared so that the area of the tooth showing caries was visualized in a plane field. This is achieved by trimming the tooth perpendicular to the occlusal areas of interest via a model trimmer continuously cooled by a flow of water where the factory-installed water solenoid automatically activates water flow and a high-performance balanced, studded diamond wheel (Unident, TBS India telematic and biomedical services Pvt Ltd.). This was guided by reference lines drawn over the tooth and the dental plaster. Trimming was performed within 40–60 s by passively holding the tooth against the diamond wheel. This technique avoids irregularities in the cut surface. The sections were then manually polished with pumice. The gross tooth sections were then placed under a microscope (OLYMPUS Binocular CH-20i Research Microscope, Mumbai, India), and the tooth surface showing the caries was visualized under reflected light. Each section was scored histologically with respect to depth under 40x magnification. The carious lesions were classified by an expert oral pathologist using Downer’s histological criteria as the gold standard (Table 2 and Fig. 1) [15]. According to binary histological criterion 1 (H1), where the threshold for treatment was low, a score of 2–4 was considered to indicate the need for active restorative treatment. In the case of criterion 2 (H2), i.e., the high-threshold criterion, the cutoff limit for restorative treatment was set at scores 3 and 4 (Table 2).

**Table 2:** Histological scoring criteria for carious lesions.

Score	Criteria	Histological low treatment threshold (H1)^*^	Histological high treatment threshold 2 (H2)^*^
0	No enamel demineralization	No intervention	No intervention
1	Enamel demineralization limited to the outer 50% of the enamel layer		
2	Demineralization extending from the inner 50% of the enamel to the dentin-enamel junction (DEJ)	Active intervention	
3	Demineralization affecting the outer 50% of the dentin		Active intervention
4	Demineralization involving the inner 50% of the dentin

### Statistical analysis

Both the ICDAS and histology scores were recorded in an Excel spreadsheet (version 2212, build 15928.20216). SPSS Version 20 (SPSS, Inc., Chicago, IL, USA) was used for statistical analysis. The inter-examiner reproducibility of the ICDAS scores for permanent posterior teeth was assessed using the kappa–Cohen statistical test. A kappa value greater than 0.75 indicates excellent agreement, whereas a value between 0.40 and 0.75 indicates fair to good agreement, and a value less than 0.40 indicates poor agreement beyond chance [16]. The sensitivity and specificity of the ICDAS with low and high treatment thresholds using low and high magnification in comparison with the gold standard histological criteria were assessed. The ICDAS binary scores obtained were compared with the gold standard as a 2 × 2 table to obtain sensitivity, specificity, positive predictive value, negative predictive value, and diagnostic accuracy via specific formulas [17]. The McNemar test was performed to compare the dichotomized ICDAS II scores with the gold standard histological scores at both low and high treatment thresholds. A *P* value of <0.05 was considered significant.

## Results

Inter-examiner reliability at ×2.5 magnification for the ICDAS-II score among examiners trained with the e-learning tool was found to be poor, with a kappa value of 0.338. However, when applying a lenient binary criterion with both low and high treatment thresholds, the agreement was very good (κ = 0.791) and good (κ = 0.706), respectively. This pattern persisted even at high magnification, where agreement was poor for the strict criteria (κ = 0.273) but excellent for the binary criteria with low thresholds (κ = 0.797) and good with high thresholds (κ = 0.727) (Table 3).

**Table 3:** Measurement of agreement between examiners via the kappa–Cohen test.

Magnification	Scoring criteria	Kappa value	Asymp. Std. Error^a^	Approx. T^b^	Approx. Sig.	*P* value
2.5X	ICDAS-II scores (0–6)	0.338	0.092	4.768	<0.001	<0.001
	Binary score criterion 1 (I1)- low treatment threshold	0.791	0.099	4.943	<0.001	<0.001
	Binary score criterion 2 (I2)- high treatment threshold	0.706	0.122	4.416	<0.001	<0.001
25X	ICDAS-II scores (0–6)	0.273	0.087	4.315	<0.001	<0.001
	Binary score criterion 1 (I1)- low treatment threshold	0.797	0.094	5.082	<0.001	<0.001
	Binary score criterion 2 (I2)- high treatment threshold	0.727	0.112	4.604	<0.001	<0.001

N of Valid Case: 39.

^a^Not assuming the null hypothesis.

^b^Using the asymptotic standard error assuming the null hypothesis.

**Fig. 2:**
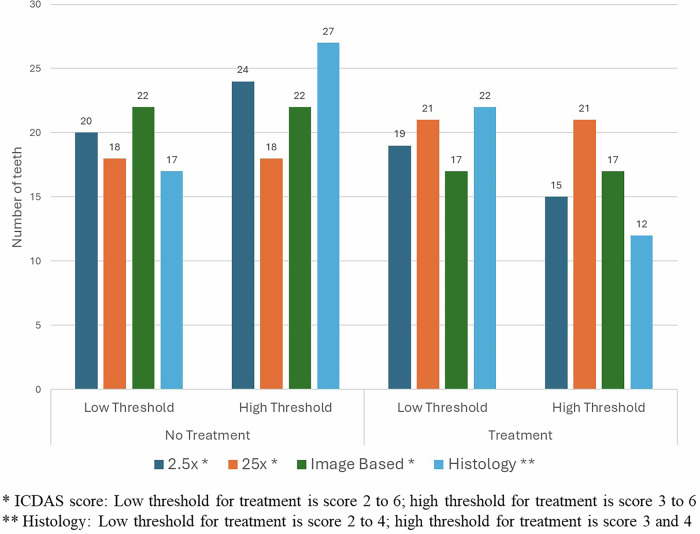
Consolidated binary scores based on the threshold for treatment.

The consolidated scores of the 39 teeth examined under various parameters are detailed in Table 4. Among the 40 samples, one sample was excluded because it was destroyed during the sectioning process. The number of teeth requiring no or active treatment, on the basis of the magnification used, expertise, and histology, is depicted in Fig. 2. When a high threshold was considered for both the histological (H2) and ICDAS binary (I2) criteria, the sensitivity was found to be highest under ×25 magnification, reaching 91.7%. Conversely, the specificity was highest under ×2.5 magnification for both low and high threshold criterion. Notably, the diagnostic accuracy was highest for ×2.5 magnification (Table 5). Additionally, image-based ICDAS scoring by an expert demonstrated good diagnostic accuracy, though it was slightly lower than the accuracy of scoring performed under ×2.5 magnification by examiners recently trained through the ICDAS interactive e-learning program.

**Table 4:** Consolidated ICDAS-II and histological scores.

Evaluation criteria	Score	Count	Column N %
2.5x loupe	Score 0	7	17.9%
	Score 1	13	33.3%
	Score 2	4	10.3%
	Score 3	6	15.4%
	Score 4	2	5.1%
	Score 5	2	5.1%
	Score 6	5	12.8%
25x operating microscope	Score 0	5	12.8%
	Score 1	13	33.3%
	Score 2	4	10.3%
	Score 3	8	20.5%
	Score 4	1	2.6%
	Score 5	3	7.7%
	Score 6	5	12.8%
Image-based ICDAS by expert	Score 0	17	43.6%
	Score 1	5	12.8%
	Score 2	0	0.0%
	Score 3	10	25.6%
	Score 4	0	0.0%
	Score 5	3	7.7%
	Score 6	4	10.3%
Histology	Score 0	11	28.2%
	Score 1	6	15.4%
	Score 2	10	25.6%
	Score 3	4	10.3%
	Score 4	8	20.5%

**Table 5:** Specificity, sensitivity, and accuracy of the ICDAS-II compared with those of histology.

	H1 and I1			H2 and I2		
**Parameter**	2.5X	25X	Image-based ICDAS by expert	2.5X	25X	Image-based ICDAS by expert
**True negative**	15	13	15	22	17	20
**True positive**	17	17	15	10	11	10
**False negative**	5	5	7	2	1	2
**False positive**	2	4	2	5	10	7
**Sensitivity**	77.3%	77.3%	68.2%	83.3%	91.7%	83.3%
**Specificity**	88.2%	76.5%	88.2%	81.5%	63%	74.1%
**Positive predictive value**	89.5%	81%	88.2%	66.7%	52.4%	58.8%
**Negative predictive value**	75%	72.2%	68.2%	91.7%	94.4%	90.9%
**Diagnostic accuracy**	82.1%	76.9%	76.9%	82.1%	71.8%	76.9%

H1: No treatment @ 0, 1; I1: No treatment @ 0, 1- Low treatment threshold.

H2: No treatment @ 0, 1, 2; I2: No treatment @ 0, 1, 2- High treatment threshold.

**Fig. 3:**
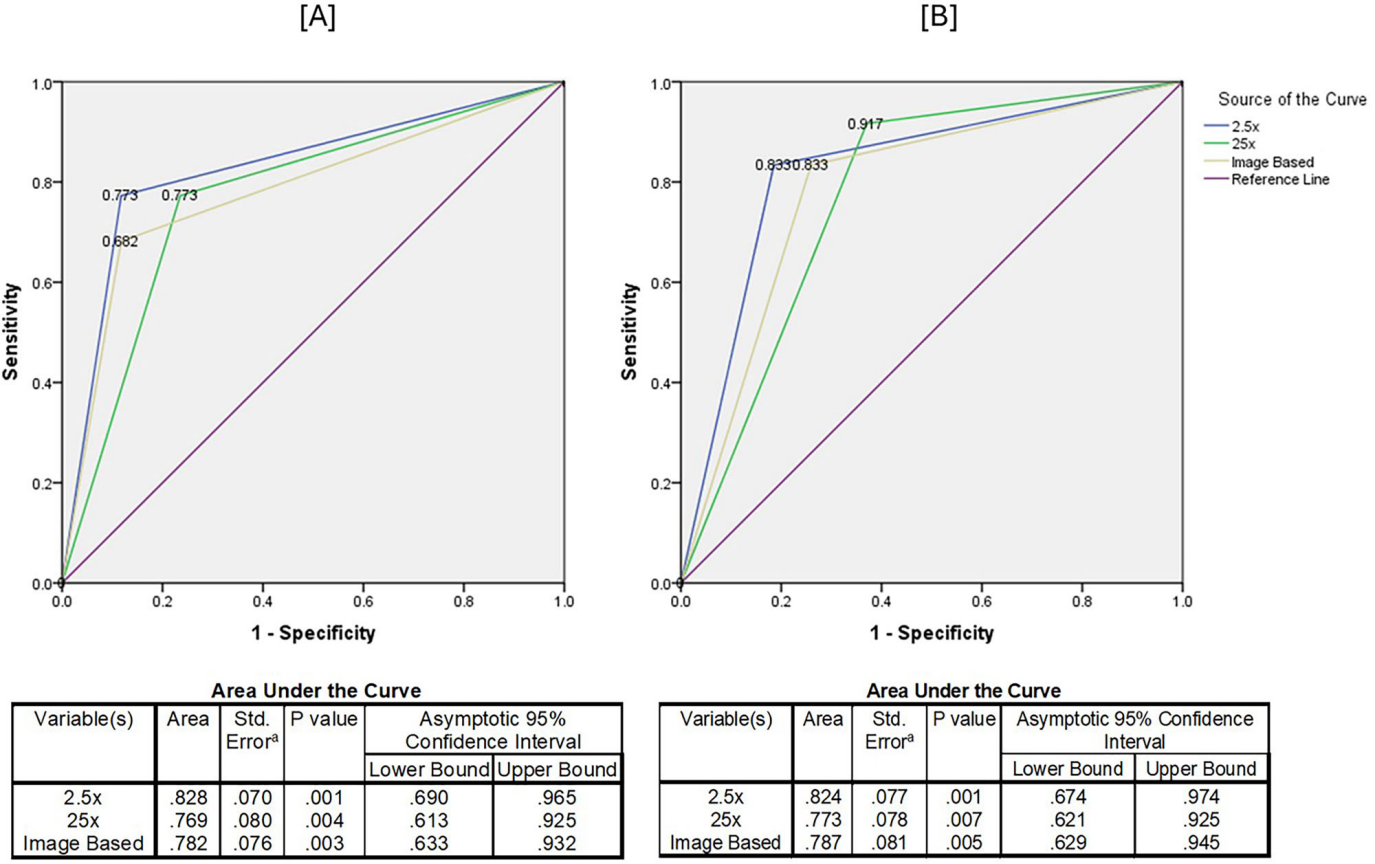
ROC curves and corresponding statistical summaries for two visual methods assessed against histological criteria. **A** corresponds to Criterion 1 (low treatment threshold), and **B** to Criterion 2 (high treatment threshold). Each panel displays an ROC curve along with a table summarizing the Area Under the Curve (AUC) and related statistical measures for key variables.

When evaluating the ROC curve, the same trends were observed, with the area under the curve (AUC) being highest for the 2.5× loupe compared to both the microscope and image-based assessments at both low and high thresholds (Fig. 3). This suggests that lower magnification provides better accuracy than extreme magnification. Additionally, there was a statistically significant disagreement between treatment needs suggested by histology and ICDAS scoring under the microscope using lenient criteria (*P* = 0.012) (Table 6).

**Table 6:** Agreement between histological and visual examination methods in determining whether treatment was required.

Mode of visualization	Need for intervention	No treatment	Treatment	
ICDAS Criterion 1 (I1)*		Histology Criterion 1 (H1)*		*P* value
2.5x	No Treatment	15	5	0.453
	Treatment	2	17	
25x	No Treatment	13	5	1.000
	Treatment	4	17	
Image Based	No Treatment	15	7	0.180
	Treatment	2	15	
ICDAS Criterion 2 (I2)*		Histology Criterion 2 (H2)*		
2.5x	No Treatment	22	2	0.453
	Treatment	5	10	
25x	No Treatment	17	1	**0.012**
	Treatment	10	11	
Image Based	No Treatment	20	2	0.180
	Treatment	7	10	

Criterion 1: Strict criteria—low treatment threshold; Criterion 2- Lenient criteria- high treatment threshold.

The bolded *P* value (*P* < 0.05) signifies statistical significance.

## Discussion

Dental caries frequently targets the occlusal surfaces of molars, which is a significant concern in dental health. Timely detection of these lesions is paramount to prevent the need for extensive and intricate treatments. Misidentification of caries lesions can result in unnecessary tooth preparation and financial burden for the patient [18]. Thus, ensuring precision in carious lesion detection is imperative for the overall well-being and satisfaction of patients [19]. Hence, the present study was conducted to detect caries at all stages so that appropriate treatment can be provided.

There are several devices available for detecting early dental caries, including those based on transillumination, fluorescence, and electrical conductance. However, these methods are often technique sensitive and expensive. Moreover, their routine use in clinical practice requires additional validation and investigation. As a result, visual examination remains the standard approach for caries detection, particularly for identifying pit and fissure caries [18]. A literature review revealed that the currently employed WHO visual scoring systems, which use DMFT scores, primarily identify cavitated lesions. However, studies have demonstrated that the ICDAS offers good reproducibility and accuracy in detecting occlusal caries as well as assessing their depth, regardless of whether the lesions are cavitated [20, 21]. However, from a clinical perspective, the inclusion of inactive lesions in the ICDAS classification is also a matter of concern because it can affect treatment decisions [22]. Hence, in the present study, we used the ICDAS criteria for detecting and assessing the progression of caries.

In this study, to mitigate bias, two examiners who had undergone training via the ICDAS interactive e-learning program before assessing carious lesions were recruited. The inter-examiner reproducibility of the ICDAS scores, regardless of the magnification used, was found to be moderate according to the kappa test. However, reproducibility significantly improved when a lenient scoring criterion was applied. This confirms that the online training module for the ICDAS effectively educates dental clinicians in comprehending the scoring criteria. This finding aligns with previous studies conducted by El-Damanhoury et al. [23] and Diniz et al. [24], which demonstrated that the ICDAS II e-learning program notably enhances the caries diagnostic abilities of dental students as well as graduates. While past research has indicated the reliability and reproducibility of the ICDAS, regardless of prior clinical experience [10, 23, 24], our study uniquely incorporated the clinical experience and knowledge of graduates in utilizing a surgical microscope, considering the steep learning curve associated with high magnification.

Interestingly, the reliability among examiners was better when a low threshold for treatment was considered for both magnifications tested. Interestingly, when there was a lower threshold for treatment across different magnifications, there was better agreement among examiners regarding reliability. This could imply that a more proactive approach to treatment might lead to clearer consensus among professionals on what actions to take, potentially reducing ambiguity or subjective interpretation. This highlights the importance of establishing standardized guidelines for treatment in diagnostic contexts to improve consistency and reliability in assessments. For enamel caries to be visible as white spot lesions after air drying, the depth of demineralization must exceed 400 μm [25]. Therefore, for an ICDAS-II score of 2, where the enamel lesion is clearly visible even when wet, the depth of demineralization will be greater than 400 μm. Hence, noninvasive remineralization therapy may be ineffective, especially in individuals with moderate to high caries risk [26]. Therefore, further studies are needed to recommend a comprehensive treatment protocol for non-cavitated enamel carious lesions and to determine the treatment threshold to be applied.

In the present study, the histological evaluation suggested by Downer was used as a control for assessing the extent of dental caries. The Downer criteria are considered the gold standard for assessing the accuracy of visual examination under various magnifications because they are easy to understand and simple to use [27, 28]. However, the main concern with histological sections is the inability to view caries in multiple planes [29]. To overcome this, nondestructive methods such as micro-CT-based in vitro studies can better classify caries in multiple planes [30]. Therefore, additional studies utilizing micro-CT as the gold standard may provide more insight into its diagnostic accuracy in caries detection.

In the present study, photographs of the occlusal surfaces of the teeth were captured and assessed using the ICDAS system by an expert with over eight years of experience. These assessments served as a reference for comparison with direct scoring performed under magnification. Despite extensive experience, the diagnostic accuracy of indirect visual methods was not superior to that of visual examination done by novice done directly under magnification. This was particularly evident for codes 0, 1, and 2, as they are recognized as the most challenging to diagnose owing to their reliance on subtle visual changes in intact enamel, which is sensitive to the moisture content of the teeth [18]. Consequently, indirect visual scoring can be unreliable even with an experienced examiner, cannot be considered a viable option. This finding contrasts with the findings of the study by Dhanavel et al. [31], which demonstrated that indirect visual examination had high sensitivity for detecting ICDAS codes 1 and 2. The difference in outcomes could be attributed to the use of the Soprocare intraoral camera for scoring in their study.

Restorative intervention is often needed when caries reaches the dentin. Therefore, in the high-threshold intervention group, the cutoff for operative treatment was set at 3 for both the histological and ICDAS-II assessments [32]. There can be clinical situations where early intervention is needed, especially for individuals with moderate to high caries risk. Therefore, in the present study, a low threshold group was introduced, with an intervention cutoff score of 2.

The level of magnification is said to influence the ability of the operator to detect minute changes on the tooth surface. Hence, we compared 2 different magnifications, 2.5x (entry-level magnification) and 25x (extreme magnification). Our study revealed that the overall diagnostic accuracy at higher magnification (25×) was lower than that at lower magnification (2.5×), with a greater incidence of false positives. Previous studies have compared the influence of magnification on the ability of the operator [5, 33, 34]. The influence of various levels of magnification, ranging from 2x to 10x, on the diagnostic outcome when the ICDAS criteria are used has been studied. However, there are no studies on the diagnostic accuracy of extreme magnification above 25x when assessing occlusal caries on the basis of the ICDAS classification.

The binary criterion uses a low threshold for early detection of caries, whereas the high threshold is for early restorative management of caries. Regardless of the caries threshold considered, the use of higher magnification did not provide any added benefit in detecting dental caries in our study. However, the sensitivity was slightly better than that at 2.5x magnification. Our present study is in agreement with a previous study by Neuhaus et al. [5], where too high of a magnification can overestimate the presence of caries. These findings have significant implications for clinical decision-making. Over-reliance on extreme magnification could contribute to unnecessary restorative procedures, increasing costs for patients and leading to the premature removal of tooth structure. Conversely, using an appropriate level of magnification, such as 2.5× loupes, can enhance diagnostic reliability while minimizing the risk of overtreatment.

A key limitation of this study is that it does not account for clinical challenges such as saliva, lighting variations, and other external factors. Therefore, the findings should be validated through further clinical studies to assess their relevance in practical settings. Additionally, future research could focus on evaluating the diagnostic accuracy of the ICDAS-II scoring system for detecting smooth surface caries in both primary and permanent teeth to expand its clinical applicability.

## Conclusion

Considering the limitations of the present study, the following conclusions can be drawn:

- Visual examination of occlusal caries using ICDAS II criteria provides a precise and valid correlation with the histological depth of caries. Hence, it is a reliable method for assessing all stages of carious lesions.
- The ICDAS-II e-learning program is efficient in teaching or training individuals to understand the coding system of the ICDAS-II.
- The cutoff value for evaluating the diagnostic accuracy of the coding system can influence the results.
- Using very high magnification for coding occlusal caries can lead to the overestimation of dental caries lesions requiring intervention.
- This study recommends a magnification of ×2.5 for caries detection and diagnostic accuracy for appropriate treatment planning.

## Data Availability

The datasets supporting the findings of this study are not publicly available. However, they can be obtained upon request by contacting the corresponding author.
